# Recurrence of a carcinoid tumor of the ovary 13 years after the primary surgery: A case report

**DOI:** 10.3892/ol.2013.1530

**Published:** 2013-08-16

**Authors:** YASUAKI AMANO, MASAKI MANDAI, TSUKASA BABA, JUNZO HAMANISHI, YUMIKO YOSHIOKA, NORIOMI MATSUMURA, IKUO KONISHI

**Affiliations:** Department of Gynecology and Obstetrics, Graduate School of Medicine, Kyoto University, Kyoto 606-8507, Japan

**Keywords:** carcinoid of the ovary, recurrence

## Abstract

The current study presents the case of a patient with a recurrent carcinoid tumor of the ovary, 13-years after the primary surgery. The primary surgery consisted of a total abdominal hysterectomy and bilateral salpingo-oophorectomy for a left ovarian tumor at 54 years old. Pathologically, the tumor was diagnosed as a carcinoid tumor of the ovary. Following the primary treatment, the patient was admitted to a cardiologist due to carcinoid-induced heart failure. At 67 years old, the patient was referred to Kyoto University Hospital with a solitary mass 8 cm in diameter and located in the paraaortic area, which was detected by routine ultrasonography and subsequent computed tomography (CT) scans. Urinary 5-hydroxyindole acetate (5-HIAA), a serotonin degradation metabolite, was present at elevated levels. With a diagnosis of a recurrent carcinoid tumor, the patient underwent a tumor resection. The pathological diagnosis was that of lymph node metastasis of the trabecular carcinoid. Post-operatively, the 5-HIAA levels returned to normal. Carcinoid tumors occasionally recur following surgery due to borderline malignant potential. Due to the slow growing nature of these tumors, in specific cases, recurrence occurs following a long interval. Therefore, a relatively long follow-up period is required.

## Introduction

Carcinoid tumors of the ovary are uncommon neoplasms. A carcinoid tumor is defined as a slow-growing neuroendocrine tumor that usually appears in the gastrointestinal tract. The unique complications derived from the secretion of serotonin are known as carcinoid syndrome, characterized by facial flushing, diarrhea, abdominal cramping, bronchoconstriction and heart failure (1–3). Primary carcinoid tumors of the ovary were first described by Stewart *et al* in 1939 and constitute 0.5–5% of all carcinoid tumors and <0.1% of all ovarian malignancies (4–7). Although the majority of ovarian carcinoid tumors are diagnosed at an early stage and are generally cured with surgical removal alone, specific cases have been reported to undergo recurrence following a number of years (8). The present study reports a case of recurrent carcinoid tumor of the ovary presenting typical features of carcinoid heart disease 13-years after the primary surgery.

## Case report

A 67-year-old female was referred to Kyoto University Hospital with a diagnosis of a paraaortic mass, identified by an ultrasound at an internal medicine clinic. This study was approved by the ethics committee of Kyoto University, Kyoto, Japan. Informed consent was obtained from the patient.

At 54 years old, the patient visited a gynecology clinic with complaints of lower abdominal distention and pain. An ultrasound identified a left ovarian tumor. Laboratory tests revealed elevated serum CA125 (95 IU/ml) levels and normal CEA and CA19-9. The patient was also diagnosed with heart failure. The individual underwent a total abdominal hysterectomy and bilateral salpingo-oophorectomy. The pathological diagnosis was of a trabecular carcinoid tumor of the left ovary with positive ascites cytology. The patient then underwent one cycle of intraperitoneal chemotherapy and three cycles of systemic chemotherapy (detailed information on the treatment was not available). The post-operative serum levels of serotonin and urinary 5-hydroxyindole acetate (5-HIAA) were normal. The heart failure was diagnosed as heart carcinoid disease. The patient was treated with medication prescribed by the cardiology department and ceased attending any gynecology appointments.

**Figure 1:**
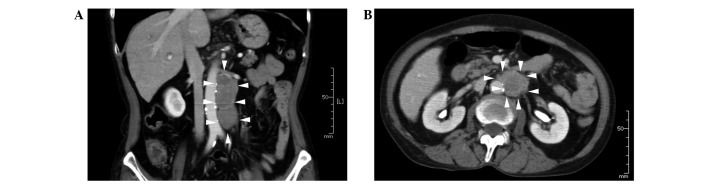
Contrast computed tomography (CT) images using lopamiron multiplanar reconstruction. (A) Colonal plane at the level of the aorta. (B) Horizontal plane at the level of the kidney veins. CT examination revealing an abnormal bulky tumor pressing on the left kidney artery along the left side of the aorta between the left kidney artery and the bifurcation of iliac arteries.

**Figure 2:**
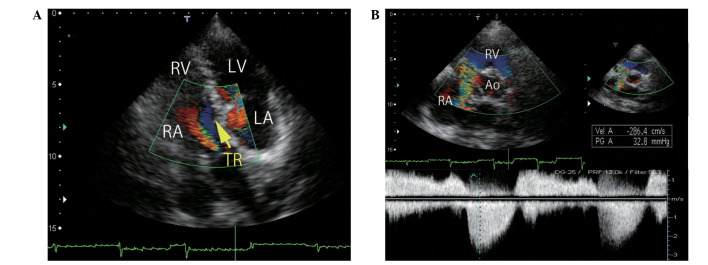
Echocardiographic imaging. (A) Color Doppler imaging of the four-chamber view reveals severe tricuspid regurgitation. (B) Continuous Wave Doppler revealing dagger-shaped curves with an early systolic peak velocity (2.9 m/s) and a rapid decline. RA, right atrium; RV, right ventricle; LA, left atrium; LV, left ventricle; TR, tricuspid regurgitation.

At 67 years old, an abnormal paraaortic tumor was identified by ultrasound screening at an internal medicine clinic, at which point the patient was referred to hospital. The patient did not complain of any typical carcinoid syndrome symptoms, which include skin flushes, diarrhea, abdominal cramping, peripheral edema or tachycardia. A computed tomography (CT) examination revealed a solitary tumor 30×30×77 mm in size in the paraaortic area (Fig. 1), while there were no abnormal observations in the pelvic cavity. Laboratory tests revealed normal serum CA125, CEA and CA19-9 levels, but the urinary 5-HIAA level was elevated to 27.5 mg/l. Consequently, the mass was diagnosed as a recurrence of an ovarian carcinoid tumor. Echocardiographic imaging revealed severe tricuspid regurgitation (Fig. 2) and mild aortic regurgitation with a pressure half-time of 655 ms. The ejection fraction was 66.5%.

**Figure 3:**
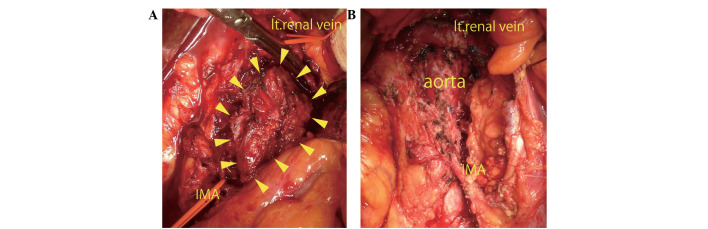
Laparotomy observations. (A) Separated left renal vein and internal mesentric artery (IMA) from the tumor are marked with vessel tape. (B) Tumor resection from the surface of the aorta.

**Figure 4:**
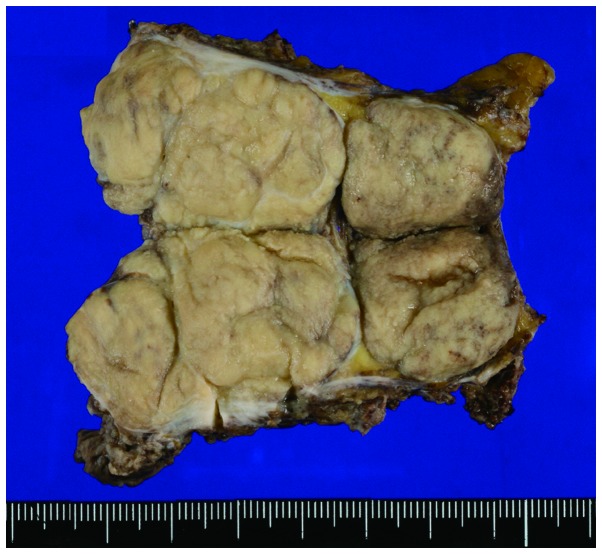
Resected tumor (paraaortic lymph nodes). Tumor size was 3×8 cm. Capsuled yellow and elastic hard tumors form specific nodules. Histological diagnosis was a diffuse carcinoid tumor spreading to the lymph node. The carcinoid type was hypothesized to be trabecular or insular in type.

**Figure 5:**
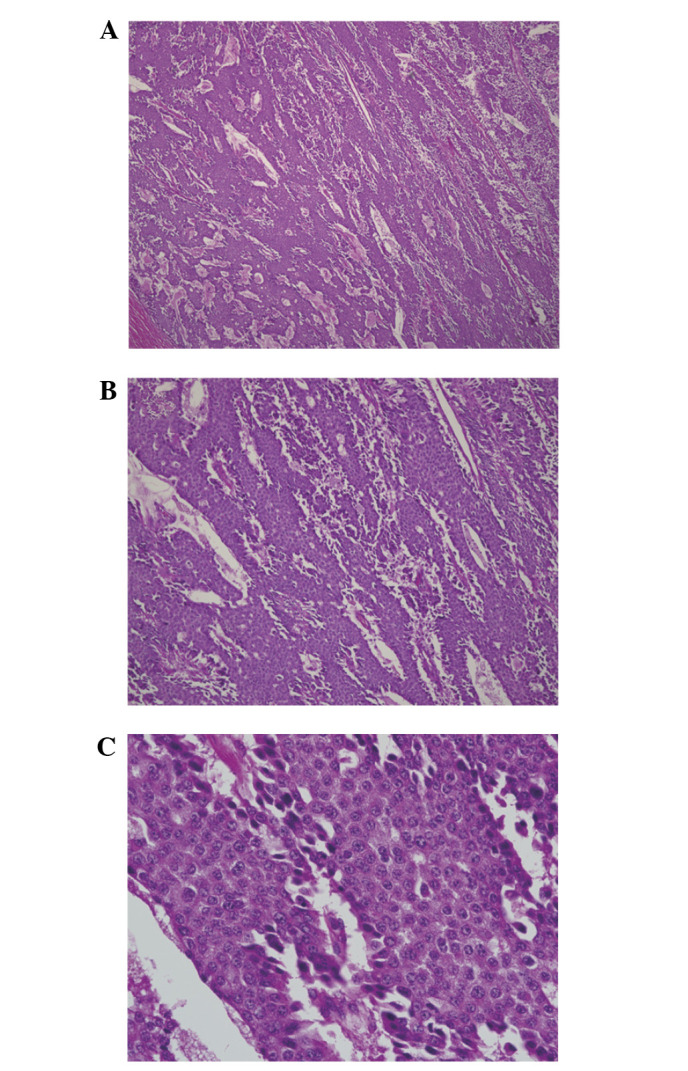
Microscopic appearance of tumor demonstrating a diffuse sheet-like structure and partial trabecular morphology. Neoplastic cells have rounded regular nuclei and a granular cytoplasm. Mitosis exists in only 1/10 high-power fields. Lymphovascular invasion may be observed (hematoxylin and eosin). Magnification, (A) ×40, (B) ×100 and (C) ×400.

Neither chemotherapy nor radiation is considered to be an effective for treating carcinoid tumors. Although the patient in this case had carcinoid heart disease, a surgical resection was performed (Fig. 3) based on the recommendations of a cardiologist who confirmed that the cardiac function would tolerate the surgery. The surgery lasted 8 h and 43 min and the total blood loss was 3,930 ml. A transfusion of 400 ml autologous blood, 6 units MAP, 8 units FFP and 20 units platelets was necessary. The histological diagnosis was that of a diffuse carcinoid tumor metastatic to the lymph nodes. The carcinoid tumor was characterized as trabecular or insular in type (Figs. 4 and 5). The patient recovered without any serious complications and was discharged 33 days after the surgery. No further treatment was administered.

## Discussion

Primary carcinoid tumors of the ovary are rare and constitute 0.5–5% of all carcinoid tumors and <0.1% of all ovarian malignancies (5–7). The histology of primary ovarian carcinoids is classified as insular, trabecular, strumal or mucinous carcinoid. The insular type often produces a large amount of serotonin and causes carcinoid syndrome, which is characterized by flushing of the skin, diarrhea and abdominal pain. More rarely, carcinoid syndrome presents as heart failure and bronchoconstriction (1,2).

The most common types of carcinoid tumors are derived from the intestines and seldom cause carcinoid syndrome since liver enzymes rapidly inactivate the vasoactive substances produced by the tumor (1,9). By contrast, primary ovarian carcinoid tumors release serotonin or other vasoactive substances directly into the systemic circulation and readily cause carcinoid syndrome, including carcinoid heart disease (?). Chaowalit *et al* previously reported 4 cases of ovarian carcinoid, which presented signs of right-sided heart failure and required surgical replacement of the valve on the right side (9). In patients with primary ovarian carcinoid tumors, ~1/3 may develop carcinoid heart disease at an early stage without evidence of metastasis. Generally, the heart failure caused by a carcinoid tumor is characterized by isolated, severe tricuspid regurgitation without significant left-sided valve dysfunction (10–12). In this case, the patient suffered from carcinoid heart disease, although the patient did not require surgical treatment. Therefore, the recurrence of the carcinoid tumor, with increased levels of serum serotonin, may have further impaired the patient’s cardiac function, which was one of the reasons for selecting a surgical resection of the tumor.

Following the primary treatment, the patient did not receive regular follow-ups with a gynecologist concerning the carcinoid tumor. However, patients with carcinoid tumors, and particularly those with cardiac dysfunction, should see a gynecologist and a cardiologist for the early detection of any possible recurrence. No effective treatment exists for carcinoid tumors, with the exception of surgical resection. Therefore, it is particularly important to detect recurrence early to ensure that surgical removal is viable and that the heart function is able to tolerate the surgery. Urinary 5-HIAA is the most reliable follow-up marker of serotonin producing-carcinoid tumors. van der Horst-Schrivers *et al* reported that persistently low urinary 5-HIAA (<20 mmol/mol creatinine) levels is a marker of a favorable survival rate (13–15). However, for detection, 5-HIAA is not as sensitive a tumor marker (specificity, 100% and sensitivity, 35%) as other markers, for example, chromogranin A (specificity, 86% and sensitivity, 68%) (16). In the case of patients who have abnormal echocardiography results at diagnosis, echocardiography every 6 months is recommended (17). In addition to the use of markers, examinations using CT scans and/or magnetic resonance imaging (MRI) is effective. It has been reported that a CT scan has 75% sensitivity and 99% specificity, while an MRI has 89% sensitivity and 100% specificity for abdominal tumor dissemination (18). Octoreotide single-photon emission computed tomography (SPECT)/CT has also been reported to be useful for detecting the metastasis or recurrence of carcinoid tumors (14). In this case, the patient was at a high risk of recurrence as the ascites cytology at the first surgery was positive. However, recurrence did not occur until 13 years post-surgery. In cases with a high risk of recurrence, particularly in patients with carcinoid syndrome at diagnosis, careful follow-up examinations must be continued for an extended period of time.
